# Acknowledgement to Reviewers of *Toxins* in 2016

**DOI:** 10.3390/toxins9010030

**Published:** 2017-01-11

**Authors:** Toxins Editorial Office

**Affiliations:** MDPI AG, St. Alban-Anlage 66, 4052 Basel, Switzerland; toxins@mdpi.com

The editors of *Toxins* would like to express their sincere gratitude to the following reviewers for assessing manuscripts in 2016. 

We greatly appreciate the contribution of expert reviewers, which is crucial to the journal’s editorial process. We aim to recognize reviewer contributions through several mechanisms, of which the annual publication of reviewer names is one. Reviewers receive a voucher entitling them to a discount on their next MDPI publication and can download a certificate of recognition directly from our submission system. Additionally, reviewers can sign up to the service Publons (https://publons.com) to receive recognition. Of course, in these intiatives we are careful not to compromise reviewer confidentiality. Many reviewers see their work as a voluntary and often unseen part of their role as researchers. We are grateful to the time reviewers donate to our journals and the contribution they make. 

If you are interested in becoming a reviewer for *Toxins*, see the link at the bottom of the webpage http://www.mdpi.com/reviewers.

The following reviewed for *Toxins* in 2016:
Abarca Salat, María LourdesGomelsky, AlexPapatheodorou, PanagiotisAbeni, FabioGoni, FelixPark, Hyun-WooAbrunhosa, LuisGoodman, LauraPark, JongHoAdams, JamesGoud, BrunoPark, YoonseongAfset, JanGovindarajan, RajagopalanParker, DaneAgeitos, Jose ManuelGratz, SilviaPascale, MichelangeloAguilera, JoséGreen, BenPascual, JulioAhmad, AamirGromadzka, KarolinaPatel, AmarAhmed, FayyazGrootaert, CharlottePavagadhi, ShrutiAhmed, WarishGruber, ChristianPecoits-Filho, RobertoAktories, KlausGuerra, ManuelaPegg, Anthony E.Albrecht, Eric A.Guerre, PhilippePeigneur, SteveAldámiz-Echevarría, LuisGulbins, ErichPeipp, MatthiasAlfonso, AmparoGutiérrez Martín, SantiagoPekár, StanoAlgood, HollyGutiérrez-Praena, DanielPellett, SabineAli, NurshadGyun, Beob GyunPérez Luzardo, Octavio LuisAli, Syed AbidHa, Tai HwanPerkins, EdwardAllavena, RachelHaas, RainerPerna, AlessandraAllen, JeremyHaesaert, GeertPerrier, JosetteAlonso, Juan CarlosHall, Jean A.Perrone, GiancarloAltmann, Daniel M.Hall, Walter A.Petruzzi, LeonardoAmézqueta, SusanaHammes, Mary S.Pflugmacher, StephanAnadón, ArturoHan, Sang-BaePfohl-Leszkowicz, AnnieAnand, PrachiHan, Sang-MiPhillips, GregoryAnderson, Rodney U.Hang, Howard C.Piccinini, RenataAndersson, Karl-ErikHanke, Wolfgang R.L.Picelli, AlessandroAndjelkovic, MirjanaHans, GuyPietri, AmedeoAndrade, FernandoHardy, MargaretPistocchi, RossellaAndrade, María J.Hasegawa, MakotoPitt, JohnAnfossi, LauraHassan, MohamedPla, DaviniaAnniballi, FabrizioHassan, Yousef I.Platts, JamesAnraku, MakotoHatano, TsutomuPletinck, AnneleenAntonissen, GuntherHayes, FinbarrPlock, Jan A.Apostolidis, ApostolosHazane-Puch, FlorencePoget, Sebastien F.Appell, MichaelHe, KaiyuPoli, MarkArakawa, OsamuHeit, BryanPolito, LetiziaAraoz, RómuloHendrich, SuzannePoluzzi, ElisabettaArbuckle, KevinHendriksen, Peter J.M.Popoff, MichelArimitsu, HideyukiHenrich, Curtis J.Porras, AlmudenaAriño, AgustínHenriques, SoniaPossani, Lourival D.Armstrong, GlenHerzig, VolkerPosthaus, HorstArnold, Sarah E.J.Heuck, Alejandro P.Potempa, JanAsgari, SassanHildebrandt, Jan-PeterPoulain, BernardAtanasova-Penichon, VesselaHo, MengfeiPrice, Michael S.Audenaert, KrisHoagland, KylePrice, Neil P.J.Avelino, AntonioHodges, Steve J.Prieto, Ana IsabelAwad, MilenaHodgson, WaynePrince, AliceAwad, WagehaHosório, HugoProctor, Richard A.Backert, SteffenHoury, Walid A.Proft, ThomasBai, JianfaHsieh, Jer-TsongProvenzano, DanieleBailly, Jean-DenisHu, Wei-GangPrusky, DovBaker, Mark D.Hu, Wen-YangPuddick, JonathanBalestrieri, BarbaraHübner, FlorianPuel, OlivierBammens, BertHuguet, AntoinePulido, Olga M.Banack, SandraHUI, Kwai FungRabsch, WolfgangBandi, ClaudioHung, Dong-ZongRadons, JurgenBaraka, KhaledHungerford, JamesRajendran, PraveenBard, FredericHurley, James C.Ramos-Vivas, JoséBarillas, JoseHurst, Mark R.H.Ranasinghe, Shiwanthi L.Barth, HolgerHust, MichaelRash, LachlanBasak, Ajit K.Hvattum, Divon HegeRasmussen, Peter HaveBateman, EmmaHymery, NolwennRealmuto, George M.Battacone, GianniIijima, NoriakiReed, KevinBattelli, Maria GiuliaImlach, Wendy L.Reverberi, MassimoBattillani, PaolaInagaki, HidetoshiRevilla, PedroBaxter, SimonInoue, SeijiRhee, Joon HaengBeaulieu, DeniseIppoliti, RodolfoRho, Jung-RaeBeaumelle, BrunoIrwin, Paul P.Rhodes, LesleyBeloglazova, N.Isbister, Geoffrey K.Ribeiro, EdnaBenedik, MichaelJ. FitzGerald, DavidRibet, DavidBenz, RolandJackson, LaurenRicci, VittorioBerrada, HoudaJang, Young DalRichard-Forget, FlorenceBerry, John P.Jenner, RonaldRobinson, KarenBerthiller, FranzJensen, LasseRodgers, Kenneth J.Bertuzzi, TerenzioJeßberger, NadjaRodrigues, PaulaBeutin, LotharJohnson, NatalieRodriguez De La Vega, RicardoBevilacqua, AntonioJohnson, Shannon L.Rodríguez, AliciaBezrukov, SergeyJordan, KieranRodríguez-Carrasco, YelkoBigner, DarrelJoshi, BharatRodriguez-Valle, ManuelBilliald, PhilippeJosic, DjuroRoelants, KimBingham, Jon-PaulJost, Wolfgang H.Rood, Julian I.Bizzarri, MarianoJuan, CristinaRosenblum, Michael G.Blanchard, Thomas G.Juan-García, AnaRosenfeld, EricBlanco, JuanKahl, Barbara C.Rosier, Peter F.W.Blangy, AnneKakabakos, Sotiris E.Rossi, FilippoBlasi, JuanKalb, Suzanne R.Roth, MonicaBöhm, JosefKaman, WendyRothkoetter, Hermann-JosefBondy, GenevieveKamitani, ShigekiRoupioz, YoannBorges, AnabelaKang, Hwan-GooRoze, Ludmila V.Bosland, Maarten C.Kang, Jin-HanRozhon, WilfriedBosso, LucianoKang, Sun ChulRubert, JosepBotana, Ana M.Karlovsky, PetrRubio, SoledadBotana, Luis M.Katikou, PanagiotaRuiu, LucaBouaïcha, NoureddineKawakami, KojiRuiz De Escudero, InigoBougis, PierreKawano, MitsuokiRumbeiha, WilsonBoujtita, MohammedKeil, KimberlyRusso, PasqualeBourne, Christina R.Kellenberger, StephanRychlik, MichaelBowers, Erin L.Kerkis, IrinaSabatier, Jean-MarcBoyer, Anne E.Kersten, SusanneSachkova, MariaBraber, SaskiaKim, BumseokSainis, IoannisBrady, RebeccaKim, EuikyungSaito, HideyukiBrandon, DavidKim, Ki-deokSakudo, AkikazuBrantl, SabineKim, Sung OukSalameh, MohammedBraun, VolkmarKim, Sung WooSalkinoja-Salonen, MirjaBraunbeck, ThomasKim, Young HoSalomonsson, MatildaBreves, GerhardKimura, MakotoSalud Bea, Roberto De LaBrigotti, MaurizioKini, ManjunathaSantamaría, RamónBrisinda, GiuseppeKiris, ErkanSantini, AntonelloBrodersen, Ditlev E.Kitamura, NaokiSatake, MasayukiBrown, AngelaKlaus, KlarskovSato, RyoichiBrown, RobertKlegeris, AndisSatta, Cecilia T.Bruce, ShenkerKleinteich, JuliaSauka, DiegoBrussow, HaraldKluess, JeannetteSavoie, Jean-MichelBryden, WayneKnight, AnthonySawa, TeijiBumann, DirkKoh, Young HoSberveglieri, VeronicaBurtey, StéphaneKoizumi, AkioSchatzmayr, GerdBusman, MarkKoizumi, SchuichiSchaumburg, FriederBuzina, WalterKollewe, KatjaScheib, HolgerCaboni, PieluigiKomatsu, MasaharuSchelin, JennyCai, ShuoweiKonno, KatsuhiroSchena, Francesco P.Calvete, JuanKonoki, KeiichiSchmidt, GudulaCamean, AnaKool, JeroenSchmidt-Heydt, MarkusCapriotti, AnnaKorch, Shaleen B.Schoevers, Eric J.Carmeli, ShmuelKormas, KonstantinosSchroeder, Christina I.Carrano, Carl J.Korotkov, Konstantin V.Schuster, Christopher F.Cary, JeffKotloski, RobertSchwartz, HeidiCasewell, Nicholas R.Kouadio, James HalbinSchwarzenberger, AnkeCasewell, NicolasKovbasnjuk, OlgaSchwedhelm, EdzardCastell, José V.Krantz, Bryan A.Schwerdt, GerladCastellano, FlaviaKudryashov, Dmitri S.Scrano, LauraCentonze, DiegoKuldau, GretchenSeidel, MichaelCerasino, LeonardoKummerli, RolfSeifert, RolandCerca, NunoKunz, WolframSelbo, Pål KristianCha, Jeong-HeonKurasaki, MasaakiSella, LucaChan, ThomasKurosawa, ShinichiroSerra, RaffaeleChanda, AnindyaKushiro, MasayoSerrão, José EduardoChang, Kung-ChaoLacy, BordenSesardic, DorotheaChartier-Kastler, EmmanuelLaganà, AldoSestili, PieroChatterjee, ChiradipLai, Chih-HoSeubert, Erica L.Chatterjee, SomLai, Wing-FuShahbazi, Mohammad AliChatziefthimiou, Aspassia D.Lambeau, GérardShama, GilbertCheli, FedericaLampert, AngelikaShan, XueyanChen, TianbaoLang, Mark L.Sharma, AtulChen, Zhi-YuanLangley, Ricky L.Shimada, TsutomuCheng, Luisa W.Lansley, SallyShin, Hyun-JaeChiesa, FrancescoLattanzio, Veronica Maria TeresaShin-ya, KazuoChijiwa, TakahitoLausten, AndreasSikorska, HannaChin, YanniLe Hégarat, LudovicSilberstein, StephenChitalia, VipulLeclercq, GuySilva, AnjanaCho, Sung-DaeLee, Bong-JinSilva, Christopher J.Cho, YukoLee, Hyang BurmSimon, StephanieChoi, Shin SikLee, John HwaSimoni, JanChoo, Myung-SooLee, Moo-SeungSingh, Kumar SaurabhChopra, Ashok K.Lee, Wing-keeSirich, Tammy L.Chou, KarenLegros, ChristianSmietana, MichaelChoudhury, DevasmitaLehman, PeggySnow, Nicholas H.Chrapusta, EwelinaLeistner, EckhardSolfrizzo, MicheleChristie-Oleza, JosephLemichez, EmanuelSonesson, AndreasCirés, SamuelLemmens-Gruber, RosaSong, JeongminCohen, GeraldLencer, WayneSorg, JoeColinet, DominiqueLeong, JohnSoto, JulioCollett, Mark G.Leonhardt, Ralf M.Soulage, ChristopheComte, KatiaLerman, Imanuel R.Soutourina, OlgaCondon, CiaránLever, MichaelSparks, DarrellCook, DanielLevesque, CelineSpicer, Leon J.Cordero, PaulLevy, HaimSpiteller, PeterCórdoba-Diaz, ManuelLiabeuf, SophieSplivallo, RichardCortinovis, CristinaLibersat, FredericSpooner, RobertCorver, JeroenLilywhite, HarveyStanker, Larry HenryCosme, FernandaLim, CheeSteffen, MorganCosta, Pedro ReisLimón, M. CarmenStegelmeier, Bryan L.Cote, ChristopherLin, Chin-YoStegmayr, BerndCox, Russell J.Lin, Hsu YangStępień, ŁukaszCrabtree, JeanLindstrom, Jon MartinSterling, TracyCraig, MorrieLinz, BodoStessl, BeatrixCreamer, RebeccaLinz, John E.Stevenson, BradleyCrickmore, NeilLlewellyn, Lyndon E.Stiles, BradleyCristofori-Armstrong, BenLoboda, AgnieszkaStiles, Bradley G.Cromwell, IanLomonte, BrunoStoll, DominicCruz, FranciscoLondon, ErwinSu, Neil QiangCummings, BrianLong, PaulSu, XinCunha, SaraLópez de Cerain, A.Sugita-Konishi, YohshikoDa Silva, Ana MaríaLópez, Carmen BlancoSugiura, KeijiDagda, Ruben K.López, PatriciaSukenik, AssafDall’Asta, ChiaraLorenz, NicoleSulyok, MichaelDaly, MaryMeganLoris, RemySumarah, MarkDaly, NorelleLosco, GiovanniSun, HongminDanforth, Teresa L.Louzao, M. CarmenSunagar, KartikDaniels-Wells, Tracy R.Luo, MengSung, Wang ChouDanneels, Ellen L.Lurling, MiguelSuntravat, MontamasDanner, Robert L.Luvisetto, SiroSusca, AntoniaDarkoh, CharlesMacrander, JasonSüssmuth, Roderich D.Dashtipour, KhashaiarMahillon, JacquesSužiedėlienė, EditaDe Baere, SiegridMahjoub, YazineSvoboda, KurtDe Bernard, MarinaMaisch, TimSwain, MartinDe Coninck, JoëlMalachová, AlexandraSycha, ThomasDe Curtis, FilippoMalissiova, EleniTadahiro, SuzukiDe Girolamo, AnnalisaMancheño, JoséTakahashi, HirokiDe Haro, LucMañes, JordiTakata, MinoruDe Kimpe, NorbertMangum, JonathanTakeda, SoichiDe Kort, Laetitia M.O.Mantle, Peter G.Takuya, KosekiDe Spiegeleer, BartMaragos, ChrisTallent, Sandra M.DebRoy, ChitritaMaresca, MarcTamagnini, PaulaDegen, GiselaMarí, FrankTartaglione, LucianaDelcour, AnneMarie, BenjaminTateo, TateoD’elios, Mario M.Marin, Jose J.G.Taylor, MatthewDell’Aversano, CarmelaMarin, SoniaTellez, GuillermoDelogu, GiovannaMarín, SoniaTerzi, ValeriaDeng, YoujunMarino, AngelaTeter, KenDeplazes, EvelyneMariottini, Gian LuigiTettamanti, GianlucaDerman, YagmurMarkaki, PanagiotaThakur, Ganesh A.Despres, LaurenceMarkland, FrankThatcher, Louise F.Dessain, ScottMarsche, GuntherThèvenod, FrankDi Masi, AlessandraMarshall, McCueThrane, UlfDias, ElsaMarte, AntonioThyagarajan, BaskaranDíaz Orejas, RamónMartinez, LuisTing, ZhouDiaz, DuarteMartinez-del-Pozo, AlvaroTisa, LouisDidier, AndreaMartinson, Ellen O.Titball, Richard W.Diego-García, EliaMärtlbauer, ErwinTittlemier, Sheryl A.Dimitrov, DimitarMaruyama, ToruTolleson, William H.Dinis-Oliveira, Ricardo JorgeMarzocco, StefaniaTolosa, JosefaDipali, SharmaMasereeuw, RosalindeTorres, JaumeDittmann, ElkeMatsui, TaeiTorres, Victor J.Dmochowski, RogerMatsuka, YoshizoTorres-Russotto, DiegoDobrindt, UlrichMattei, CesarTortorella, DomenicoDolly, OliverMattern, DerekTouchard, AxelDomashevskiy, Artem V.Mattoo, SeemaToyotome, TakahitoDong, MinMatyas, Gary R.Trainer, VeraDorantes-Aranda, Juan JoséMaynard, JenniferTrendowski, MatthewDorman, Charles J.Mazor, OhadTrim, Steven A.Dosio, FrancoMazzone, GloriaTsai, Inn-HoDossena, SilviaMcClain, MarkTsatsakis, AristidisDouthwaite, StephenMcClain, Mark S.Tsikas, DimitrosDowd, Patrick F.McClane, BruceTsitsigiannis, DimitriosDoxey, Andrew C.McCluskey, Andrew J.Tsujimoto, MasayukiDuan, WeiMcCormick, JohnTudzynski, BettinaDübel, StefanMcCormick, SusanTufféry, PierreDucancel, FrédéricMcgrath, SaraTumer, NilgunDufresne, MarieMcmorrow, TaraTurley, EvaDuringer, Jennifer M.Mcnally, AlanTurrà, DavidDutertre, SebastienMebs, DietrichTyler, BettyDuval, DavidMeca, GiuseppeTzika, EleniEaton, David L.Meijer, Harold J.G.Uchida, YoshikazuEberhart, Bich-Thuy L.Mellon, Jay E.Uchiumi, ToshioEble, JohannesMelville, StephenUegaki, RyuichiEckardstein, ArnoldMelzig, MatthiasUhlig, SilvioEckford-Soper, Lisa K.Mendell, Mark J.Ulett, Glen C.Edwards, ChristineMerrick, B. AlexUmemura, MycoEichacker, Peter Q.Mertz, Janet E.Undheim, EivindEldering, EricMetcalf, JamesVago, RiccardoEl-Shehaw, RehabMetcalf, Jordan P.Vaillant, FabriceEl-Wakeil, NabilMetz, MartinValentin-Weigand, PeterEngelberg-Kulka, HannaMetzler, ManfredValiante, VitoErickson, PeterMeulenberg, ElineVan Der Merwe, DeonEspinosa, ManuelMiedaner, ThomasVarga, ElisabethEsquenazi, AlbertoMikheyev, Alexander S.Vasconcelos, VitorEtxebeste, OierMiles, JohnVaughan, Martha MEvans, Mary AnneMiller, DavidVeeratterapillay, RajanEveraert, KarelMiller, SandraVella, Anthony T.Eyer, FlorianMin, Kyung-JinVenancio, ArmandoEymar, EnriqueMinervini, FiorenzaVenkitanarayanan, KumarFabbro, LarelleMinocha, Subhash C.Vetter, IrinaFagard, MathildeMirasoli, MaraVisagie, Cobus MeyerFahrer, JörgMirey, GladysVisvikis, OraneFalconer, IanMitchell, Dr. Thomas K.Vita, Di StefanoFanelli, FrancescaMiyashita, MasahiroVreugde, SarahFang, Evandro FeiMol, HansWagg, AdrianFaresse, NourdineMolinier-Frenkel, V.Wakino, ShuFaul, ChristianMonastyrskaya, KatiaWalker, AndrewFedak, GeorgeMontecucco, CesareWalls, ZacFei, PeiwenMontuori, PaoloWalton, Jonathan D.Feig, AndrewMoon, YuseokWan, Lam YimFels-Klerx, Van Der H.J.Moore, CatherineWang, Jia-ShengFernandes, Ana S.Moran, YehuWang, PingFernandes-Ferreira, ManuelMoretti, AntonioWang, ZenengFernández-Cruz, María LuisaMorita, KyojiWatanabe, HiroshiFerreira, Rui M.Mou, XiaozhenWeaver, KeithFerreras, José MiguelMullany, PeterWeaver, MarkFEUILOLEY, Marc G.J.Mullasseril, PraseedaWeber, BarbaraFineran, PeterMüller, AnneWeiergräber, MarcoFink-Gremmels, JohannaMuller, MichaelWelch, Aaron Z.Firestone, GaryMulley, John F.Weller, MichaelFischer, ReinhardMulo, PaulaWheeler, DaveFisher, Aron B.Mulvenna, JasonWhittington, Camilla M.Fisher, Mark T.Munaut, FrancoiseWider, GerhardFlannery, BrennaMundt, SabineWiedmann, MartinFlavell, David J.Nabok, AlexeiWiegand, ClaudiaFloriano, Wely B.Nagaoka, KentaroWiemann, PhilippFöllmann, WolframNagata, KojiWiens, JohnFong, DunneNakane, AkioWilcox, ChristieForoud, NoraNathanail, AlexisWilliams, DavidFourtounas, CostasNaumann, MarkusWillis, AnusuyaFox, JayNaumann, Todd A.Wilson, Brenda AnneFribley, Andrew M.Negro, AndreaWindham, GaryFrisan, TeresaNelson, Rebecca J.Windle, Henry J.Frisvad, JensNestorovich, EkaterinaWinkel, Kenneth D.Fry, BryanNevalainen, TimoWong, Jon W.Fujii, HidekiNguyen-Thé, ChristopheWood, SusannaFujinaga, YukakoNick, Harry S.Wood, ThomasFurlanetto Pacheco, Ana BeatrizNiculita-Hirzel, HélèneWorrell, RogerFusco, VincenzinaNiehuis, OliverWoychik, NancyGagliano, NicolettaNielse, Vance G.Wray, Kenneth P.Gajewski, Jerzy B.Niessen, LudwigWu, Sheng-NanGalat, AndraejNiu, Yan D.Wu, Tung-KungGalaverna, GianniNoda, NaohiroWuster, WolfgangGall, BrianNookala, SubaXiao, JunGallo, AntonioNorton, RaymondXue, Li.Gally, David L.Nossol, ConstanzeYabe, KimikoGámiz-Gracia, LauraNuttall, Patricia AYahiro, KinnosukeGannon, Victor P.J.O’Donnell, Michael JYamabhai, MontaropGarcía-Ortega, LucíaOchsner, Urs A.Yamada, KeikoGardner, Paul D.Oelschlägel, MichelYamaguchi, YoshihiroGareis, ManfredOgihara, JunYamashita, Mari YotsuGastaldello, StefanoOhi, Melanie D.Yarden, RonitGazerani, ParisaOkumura, ShiroYeager, Linsey A.Gazzotti, TeresaOkumura, ToshikatsuYli-Mattila, TapaniGehlsen, Kurt R.Oliveira, Carlos AugustoYogendra, KalenahalliGekle, MichaelOlombrada Sacristán, MiriamYoshinari, TomoyaGelens, LendertOnduka, ToshimitsuYoung, HeatherGenevaux, PierreOpitz, BastianYu, Feng-YihGerardo, Charles JOppert, BrendaYu, HaiGerdes, KennOrth, Joachim H.C.Yu, Hong-JengGerhard, RalfOsorio, CarlosYun, Hae KeunGerwin, RobertOswald, EricYunus, AghaGiannantoni, AntonellaOtsuka, YuichiZaccaroni, AnnalisaGibbs, H. LisleOtsuka, YuzuruZelanis, AndréGilbert, Robert J.C.Özbek, SuatZeleny, ReinhardGillet, DanielPadula, AndrewZentek, JürgenGilliam, Lyndi L.Pak, Sok CheonZhang, KaiGiovannoli, CristinaPalma, LeopoldoZichel, RanGirbés, TomásPalmer, MikeZiemer, med. habil. MirjanaGiuberti, GianlucaPanagou, EfstathiosZobel-Thropp, Pamela A.Gkelis, SpyrosPannek, JürgenZygadlo, JulioGlorieux, GrietPanza, Francesco
Goldoni, LucaPapadimitriou, Theodoti


